# Toward Rational Design of Ion-Exchange Nanofiber Membranes: Meso-Scale Computational Approaches

**DOI:** 10.3390/membranes16010005

**Published:** 2025-12-23

**Authors:** Inci Boztepe, Shuaifei Zhao, Xing Yang, Lingxue Kong

**Affiliations:** 1 Institute for Frontier Materials, Deakin University, Waurn Ponds, Geelong, VIC 3216, Australia; iboztepe@deakin.edu.au (I.B.); s.zhao@deakin.edu.au (S.Z.); 2 Department of Chemical Engineering, KU Leuven University, Celestijnenlaan 200F, B-3001 Leuven, Belgium; xing.yang@kuleuven.be

**Keywords:** protein binding, ion-exchange nanofiber membrane, computational approaches, meso-scale simulations, CFD, DEM

## Abstract

This review highlights the growing relevance of ion-exchange nanofibrous membranes (IEX-NFMs) in membrane chromatography (MC) for protein purification, emphasising their structural advantages such as high porosity, tunable surface functionality, and low-pressure drops. While the adsorption of IEX-NFMs in MC is expanding due to their potential for high throughput and rapid mass transfer, a critical limitation remains: the precise binding capacity of these membranes is not well understood. Traditional experimental methods to evaluate protein–membrane interactions and optimise binding capacities are labour-intensive, time-consuming, and costly. Therefore, this review underscores the importance of computational modelling as a viable predictive approach to guide membrane design and performance prediction. Yet major obstacles persist, including the challenge of accurate representation of the complex and often irregular pore structures, as well as limited and/or oversimplified adsorption models. Along with molecular-scale simulations such as molecular dynamics (MD) simulations and quantum simulations, meso-scale simulations can provide insight into protein–fibre and protein–protein interactions under varying physicochemical conditions for larger time scales and lower computational burden. These tools can help identify key parameters such as binding accessibility, ionic strength effects, and surface charge density, which are essential for the rational design and performance prediction of IEX-NFMs. Moreover, integrating simulations with experimental validation can accelerate optimisation process while reducing cost. This technical review sets the foundation for a computationally driven design framework for multifunctional IEX-NFMs, supporting their use in next-generation chromatographic separations and broadening their applications in bioprocessing and analytical biotechnology.

## 1. Introduction

Membrane chromatography (MC) is an alternative process used in protein purification based on affinity, ion exchange, and hydrophobic interaction separation. In MC, stacks of microporous or macroporous functionalised polymeric membranes with larger pore sizes in a range from 0.65 to 3 µm [1] are placed in the capsule as an adsorptive medium [2]. While the stationary phase is the chromatographic medium or the adsorbent, the mobile phase is the solution that flows through the column [3,4]. MC can be operated in either batch or flow-through modes. Continuous or flow-through adsorption provides higher productivity and better quality compared to batch adsorption. The transition from batch to continuous processing is relatively straightforward, requiring only minimal modifications like adjustments in column size and flow rate [5]. Higher flow rates can be used due to the convective nature of the mass transfer through membranes, resulting in almost flow-independent binding.

In the ion-exchange process, separation is governed by electrostatic interactions between proteins and charged groups on the membrane, depending on the pI value of the protein [6]; cation-exchange membranes bind positively charged proteins, while anion-exchange membranes bind negatively charged proteins [7]. Hydrophobic interaction membrane chromatography relies on interactions between hydrophobic ligands (e.g., butyl, octyl, phenyl) and non-polar regions on protein surfaces, typically under high salt conditions to exclude polar impurities [8,9,10]. Affinity membrane chromatography utilises immobilised ligands that selectively recognise and bind target proteins via specific biorecognition mechanisms [7]. Mixed-mode membrane chromatography combines multiple interaction types such as ion-exchange, hydrophobic interactions, and hydrogen bonding. A combination of ion exchange and hydrophobic interactions can usually achieve high selectivity and sensitivity [9]. However, mixed-mode chromatography media preparation usually requires a complex process and makes it difficult to control ligand density [11]. In Table 1, the most common chromatography methods for protein separation can be found [3].

The MC process has several distinctive advantages over the conventional packed column, such as high flexibility, high flow rates, low-pressure drop, scalability, and potential for high-throughput operation [15,16]. Disposable MC capsules are especially useful in small- to preparatory-scale bioprocessing as they eliminate the need for column cleaning, packing, and process revalidation, making them suitable for small-scale production in early clinical studies [17,18,19]. Also, due to the high modularity of membrane-based systems, large volumetric capacities and high velocities can be employed in MC [20,21].

The mass transfer mechanism is the distinctive factor between column and membrane chromatography. The solute transport in membrane chromatography mainly occurs through convection to the membrane’s internal surface, which is a much faster mechanism compared to the intraparticle diffusion of proteins through the pores in column chromatography [1,22]. This convective transport effect results in a much lower mass transfer resistance [22].

The main limitation to the large-scale industrial application of MC is its relatively low binding capacity [23]. This issue arises primarily from restricted access to functional sites, often caused by inadequate pore connectivity and flow maldistribution within the membrane matrix [18]. Membranes with larger pore sizes generally possess smaller volume-specific surface areas compared to those with smaller pores at the same porosity, which reduces available binding sites. Nevertheless, in some cases, non-uniform porosity, membrane thickness, and ligand grafting can lead to variable flow resistance within the porous media, which could result in early saturation of binding sites in large pores and a decrease in dynamic binding capacity. These properties should be treated carefully for the sake of maintaining the high-throughput membranes in protein purification.

To maintain high throughput in protein purification, careful control of these structural parameters is essential. Enhancing the binding capacity of adsorptive media, therefore, requires material-level innovations. Recent developments include the use of next-generation matrices such as hydrogels and nanofibres, which provide higher specific surface areas, increased ligand densities, and optimised 3D binding environments [19]. Advances in nanotechnology have further enabled the fabrication of nanofibrous membranes with interconnected pore networks and high porosity, significantly improving mass transfer and accessibility to active sites [18,19,24,25]. Owing to their exceptionally high specific surface area (~10–40 m^2^·g^−1^), ion-exchange nanofibre membranes represent a promising alternative for enhancing protein-binding performance in membrane chromatography [18].

Despite significant progress in ion-exchange nanofibre membranes for protein binding, the field still lacks a unified framework that links membrane structure, transport phenomena, and performance in a way that enables rational and predictive design. Several reviews have provided valuable insights into ion-exchange nanofibre membranes, including a comprehensive discussion on design principles, synthesis methods, and an application performance study of electrospun nanofibrous membranes [15], as well as fundamental aspects, scalable fabrication techniques, and broad application areas of IEX nanofibres [26]. More recently, reviews have provided innovative approaches for developing novel membranes with properties tailored to the future needs of downstream processing [27], detailed assessments of next-generation high-throughput ion-exchange membranes and the factors influencing their performance [1], and examined the rational selection of membrane materials and structural properties for efficient bind-elute antibody capture [28].

As the field moves toward next-generation chromatographic membranes, optimising nanofibrous pore structures and surface functionalities will be crucial to enhancing adsorption performance. A deeper understanding of how pore architecture, fibre morphology, and surface modifications affect mass transport is essential [15]. As experimentally evaluating all parameters that influence membrane performance is time-consuming and resource-intensive, computational simulations offer a powerful alternative through modelling flow, adsorption kinetics, and mass transport mechanisms and provide critical insights into guiding membrane design. These simulations are particularly valuable for understanding how pore structure and surface properties affect separation mechanisms in ion-exchange processes, directly linking membrane characteristics to protein-binding efficiency. Systematic studies of individual membrane characteristics and their impact on binding capacity remain limited in the literature. This review, therefore, aims to establish a rational design framework in which computational approaches facilitate the development of next-generation ion-exchange membranes tailored for efficient protein-binding and chromatographic applications.

## 2. Protein Adsorption on Ion-Exchange (IEX) Nanofibre Membranes

Recently, modified polymeric membranes have gained prominence in downstream processing, particularly in biological filtration units, due to their enhanced performance in protein separation [29]. Among various fabrication methods, electrospinning has attracted significant attention in protein purification for its precise control over fibre assembly and morphology [15,30,31]. Electrospun nanofibrous membranes are especially promising in ion-exchange membrane chromatography (IEMC) because of their high specific surface area (SSA), highly tortuous porous structures, and robust mechanical stability, all of which promote efficient protein adsorption [32]. Additionally, uniform nanofibres can be produced in a continuous process with control over the structure [33]. Specifically, electrospun nanofibre membranes enable chemical/physical functionalization that can result in high separation efficiencies [34]. Because of functionalization, the majority of binding sites on the surfaces of these nanofibres are accessible and external, resulting in a high adsorption capacity. This leads to numerous accessible binding sites on the nanofibres, therefore increasing the binding capacity of membranes.

Protein adsorption on the IEX nanofibre membrane occurs through static or dynamic adsorption. In static adsorption, the membrane acts as an adsorbent, with surface area being the key determinant of adsorption capacity. In dynamic adsorption, the membrane integrates adsorption and filtration within a single-pass flow [26]. Reported dynamic binding capacities (DBCs) for commercially available micron-size fibrous membranes range from 29 to 70 mg/mL bovine serum albumin (BSA) for anion exchange membranes and 29 to 47 mg/mL Lysozyme for cation-exchange membranes [1]. In comparison, electrospun nanofibre membranes demonstrate notably higher binding capacities in membrane chromatography, as summarised in Table 2.

Functional ligands are attached to the internal pore surfaces throughout the membrane structure and significantly enhance binding by increasing the number of accessible binding sites [12]. The grafting of charged or ion-exchange groups, such as carboxyl, DEAE, COO^−^, and CCA, enhances electrostatic interactions between the membrane and target proteins, thereby improving binding efficiency [16,37,40]. This functionalization significantly improves the overall binding capacity, as the modified surface provides a higher density of accessible ligands for binding. For example, PAN-pAQ and PAN-GMA-DEA membranes functionalized with quaternary amine groups achieved up to 260 mg/g static binding capacity for BSA [35,36]. Notably, pH-responsive PVDF-g-PDMAEMA (poly(vinylidene fluoride)-graft-poly(dimethylaminoethyl methacrylate) membranes with anion exchangers achieved static and dynamic binding capacities of 318 mg/g and 210 mg/g, respectively [41]. Moreover, hydrophilic modification, as reflected by lower water contact angles, tends to promote protein accessibility and enhance binding. During functionalization, fibre diameter might vary, which can cause the loss of SSA because of the direct correlation between fibre diameter and SSA. Hence, variations in fibre diameter should be monitored before and after the functionalization of the nanofibrous membranes [1].

A smooth fibre surface and uniform fibre radius can be achieved after electrospinning the membrane, favouring higher throughput of the membrane during purification of proteins [42]. Tortuous micro channels also provide more available adsorption sites for the membranes [39]. For instance, the SA-PEO membrane, with fine nanofibrous networks and moderate pore sizes (0.3–0.6 µm), achieved exceptional lysozyme-binding capacities [37]. PVA-MAH membranes with different PVA content showed improved lysozyme adsorption due to their high specific surface area (3.2–3.5 g/m^2^) and relatively low hydrophobicity, which together increase available binding sites and promote stronger protein–surface interactions [39]. These findings highlight that optimising nanofibre diameter, pore connectivity, and surface chemistry is crucial to balancing mass transfer and binding efficiency in next-generation ion-exchange membranes. In protein purification, high permeance typically requires membranes with porosity above 70%, and pores larger than 100 nm allow macromolecules to pass without affecting separation, while pore size directly influences binding capacity and permeability. Larger pores increase permeability but reduce specific surface area, whereas a narrow pore size distribution promotes uniform flow and enhances dynamic binding [1].

Future membrane fabrication for chromatographic applications should prioritise optimising nanofibrous membrane pore structures to enhance adsorption performance. Understanding pore architecture and surface modification effects on mass transport is essential for evaluating membrane-binding performance. Experimentally evaluating all parameters influencing process performance is time-consuming and resource-intensive. Computational simulations offer a powerful alternative by modelling flow and adsorption kinetics, providing insights into mass transport mechanisms and guiding membrane design. These simulations are particularly valuable for understanding how pore structure and surface properties influence the separation mechanism in ion-exchange processes, linking membrane characteristics directly to protein-binding efficiency.

### 2.1. Separation Mechanism of IEX Binding

In IEMC, the adsorption of protein on adsorbent membranes occurs via an ion-exchange mechanism. Separation and purification occur because of electrostatic interactions between the proteins and charged functional groups on the ion-exchange membrane [43]. Binding efficiency can be determined based on the nature of the proteins, adsorbents, and process conditions [44]. Ultimately, these factors affect the protein–adsorbent interactions, typically with electrostatic interactions being recognised as the primary influence. The electrostatic interaction between the adsorbent (nanofibre surface) and the adsorbate (protein) plays a crucial role in protein binding. An adsorbent surface with a charge opposite to that of the protein can be more effective at adsorption compared to one with the same charge [29]. Additionally, the magnitude of the charges plays a crucial role in determining protein binding [40,45]. Therefore, for ion-exchange membranes, protein binding depends on the balance of electrostatic attraction between the surface charge of the membrane and the protein [46].

### 2.2. Factors Impacting the Binding of Proteins on IEX Nanofibres

The adsorption mechanism of IEX nanofibres is influenced by factors related to the surface of the adsorbent, protein characteristics, and solution conditions. The surface charge of the nanofibre, as determined by its zeta potential, directly affects the strength of protein attraction towards the surface. Similarly, the charge properties of the protein, governed by its pI (isoelectric point), the point of zero net charge, and the pH of the surrounding medium indicate whether the protein is negatively or positively charged [9]. Additionally, the ionic strength of the solution plays a critical role by influencing the Debye length, which determines the range of electrostatic forces [47]. Morphological properties related to both membranes, such as specific surface area (SSA), pore size, and porosity [35], and the protein, such as the size and shape [48], can also impact the magnitude of the electrostatic interactions. The charge of the protein can be altered by adjusting pH. When the pH is below the protein’s isoelectric point (pI), the protein carries a net positive charge, whereas at pH values above the pI, the protein becomes net negative [49]. Since proteins exhibit varying electrical properties in buffer solutions at different pH levels, protein purification can be achieved by exploiting the controllable electrostatic interactions between the protein and the adsorbent [15].

#### 2.2.1. Adsorbent Structural Effect

The ligand density can be correlated to the surface charge of the nanofibres [18]. Generally, as the ligand density increases, the binding capacity of the membrane also increases, exhibiting a direct and linear correlation due to the promoted grafting area [36,50]. On the other hand, the binding capacity was compromised with ligand density content as the adsorption capacity significantly reduced with a further increase in ligand content (after 3% w% of BTCA (butane tetracarboxylic acid)) because of a decrease in the effective adsorption area. This can refer to how the ligands are arranged or interact with each other, potentially blocking or obstructing available space for binding [51]. Similarly, the ligand density increased with pH (8.5 to 13), and IgG binding capacity increased from pH 8.5 to 10. However, despite the increasing ligand density from pH 10 to 13, the IgG binding capacity decreased during this range. This was linked to the limited accessibility of IgG to densely packed ligand binding sites in the grafted layers [52]. Optimum binding can be achieved by uniform ligand distribution on the IEX nanofibres [53]. Therefore, ligand density optimisation is important to employ the modification without compromising the membrane properties, such as porosity and fibre diameter. For instance, after functionalisation of the membrane, the porosity was compromised in the range of 1.14–4.6% [50], and in most cases, pore morphology was intact after the functionalization of the IEX membranes.

Besides grafting and modification, membrane and protein morphology are also crucial. For example, pore connectivity determines how effectively the internal pore network is interconnected, which directly affects intrapore diffusion and the accessibility of binding sites [54]. Highly interconnected pore networks facilitate uniform fluid pathways, thereby enhancing membrane performance, offering practical insights for the design of next-generation materials [55]. It can be assessed in a semi-quantitative manner through techniques such as tracer diffusion, permeability measurements, or 3D imaging approaches (e.g., XCT, FIB-SEM) [56,57]. There is a complex trade-off relationship between surface micro/nano-structure and, hence, distribution and/or density of functional groups [18]. Overall, it was concluded that the high SSA of the nanofibrous IEX membrane allows a higher ligand density, thereby demonstrating a higher binding capacity. For instance, a higher adsorption capacity was reached with a 270% smaller fibre diameter and 400% higher SSA of the membrane in comparison to the study reported in [32]. Compared to the macroporous microfibres, IEX nanofibres had more binding capacity, which can underline the importance of the morphological properties of the membrane [18]. To understand the correlations between membrane structure and binding performance, various membranes were fabricated under varying electrospinning process parameters [35]. At a fixed pAQ content (15 mol%), the fibre orientation and alignment significantly influenced the protein binding capacity. Additionally, the binding with the well-oriented fibres increased by 45–66% compared to the randomly oriented fibres. Regarding protein morphology, studies found differences in binding to the membranes due to their charge differences [51]. Besides the isoelectric points and charge differences with the adsorbent surface, in some cases, the surface of the nanofibres was more favourable for specific types of proteins, indicating their distinctive nature for molecular weight [31,58].

The zeta potential of nanofibres and proteins plays an important role in ion-exchange chromatography. The overall surface charge, in any suspension, can be determined by the zeta potential values [59]. Zeta potential indicates the strength of surface charge on particles and primarily influences adsorption processes at longer time scales. The maximum adsorption was reached with the N-4ZH nanofibrous IEX membrane compared to the other membranes due to its highest zeta potential and uniform distribution of 4ZH nanoparticles within the fibres [29]. The effects of zeta potential can be explained by DLVO theory to describe the stability of the system by considering different forces acting on the particles [60]. High zeta potential, either positive or negative, is generally required to ensure stability. Thus, systems with zeta potentials > ± 30 mV are generally considered stable. The DLVO approach has been successfully used for the evaluation of biological particle adhesion in the context of bioprocessing [61]. This approach was found as appropriate to be used as the theoretical framework for protein interaction studies to predict protein behaviour during binding [61]. With DLVO theory, protein interactions with the surface can be described as colloidal representations. The DLVO theory explains that the tendency of a colloidal particle to move toward or away from a surface depends on the balance of attraction and repulsion forces acting on it [62].

#### 2.2.2. Protein Properties

Protein characteristics such as its structure (e.g., dimension, stability), size, and net charge affect the binding mechanism. The structure of proteins depends on the sequence of amino acids, and it can vary among proteins. The structural stability of proteins indicates the ease of undergoing conformational modification. Larger-sized proteins have more binding sites to interact with the surface, thereby increasing the possibility of binding to the surface. In certain instances, such as when the protein solution comprises a mixture of hundreds of different proteins, e.g., blood plasma, the mass transfer rate of protein molecules to the surface can be directly correlated with its concentration and inversely related to its molecular weight [63]. Through experimental observations, proteins that were at or near the isoelectric pH exhibited greater adsorption because of minimised charge–charge repulsion among the adsorbed molecules.

The adsorption behaviour of proteins is also controlled by the stability of a protein, which can be one of the driving forces during adsorption. The underlying reason for this is that the conformation of a folded protein is highly constrained, resulting in relatively low entropy. However, if the protein undergoes partial or complete unfolding upon adsorption, this process can lead to an increase in conformational entropy, thereby acting as a driving force. Additionally, the conformational stability of a protein plays a critical role in determining the structure of the adsorbed protein. For example, soft proteins such as bovine serum albumin (BSA) can have relatively low structural stability and undergo extensive surface rearrangement. These can promote an increased number of interaction points among the proteins and the surface and adsorbed proteins. Hard proteins such as lysozyme and Chymotrypsin can be more resilient to major changes and can primarily adsorb to hydrophobic surfaces unless electrostatically attracted [64,65,66].

Electrostatic interactions in proteins are complex due to the heterogeneous medium surrounding protein charges. While water, the solvent in aqueous solutions, is highly polar with a dielectric constant of nearly 80 at room temperature, the protein interior is largely non-polar, with a dielectric constant typically ranging from 2 to 4, though values up to 40 have been reported. Charges within the protein interact with both the solvent and other charges, with the solvent effectively screening these charge–charge interactions. These charge–solvent interactions and the solvent’s screening effect play a crucial role in shaping the electrostatic energies of proteins [67].

#### 2.2.3. Solution Parameters

The pH value of the solution, as one of the crucial parameters for the performance of protein adsorption, is a determinative factor for the surface potentials of both nanofibres and protein molecules. While a lower pH may not be ideal for some proteins’ charge magnitude for electrostatic interaction, it can be suitable for others. For example, pH 5.5 has the highest binding for IgG (pI = 7.8), whereas it can result in lower binding for BSA and lysozyme [42]. The reason behind this phenomenon is that when the pH is equivalent to 8.0, IgG is negatively charged, causing the electrostatic repulsion between the protein and the adsorption ligand. Conversely, the adsorption capacity of lysozyme increased as the pH decreased below the protein’s pI 10.8, reaching a maximum at pH 6 due to the enhanced positive charge of lysozyme and negative charges on the PMA-functionalized nanofibres at lower pH [32]. For membranes with quaternary amine groups, the nanofibre surface charge remains positive and is substantially unchanged unless exposed to unrealistically extreme pH values. In contrast, membranes functionalised with tertiary amines show a strong pH-dependent surface charge, making pH the dominant factor governing their zeta potential [41]. As a result, electrostatic interactions can be controlled with a pH-responsive IEX membrane through switchable surface charges [68]. BSA adsorption was tuned by varying pH values in the range of 3.6–9.0. BSA binding increased sharply when the pH was increased from 3.6 to 6.4 and decreased sharply after pH 6.4. This is because at pH 3.6, the BSA molecule was positively charged, resulting in charge repulsion, and at pH 6.4 the BSA molecule (pI 4.7–4.9) was negatively charged, leading to significant binding of BSA. Additionally, a pH-controllable IEX nanofibrous membrane can be used for selective protein adsorption by regulating buffer pH value based on the pI of the target protein [34]. The effect of pH on BSA colloidal stability does not directly correlate with surface charge, as it reflects the interplay between van der Waals attraction and electric double-layer repulsion [69]. The variation in zeta potential with pH was more significant at pH values above 4.7 than at pH below 4.7 for BSA [70]. Therefore, it is important to understand the impact of pH on the electrostatic interaction between the IEX nanofibres and the protein.

The ionic strength of the solution can be adjusted to maximise the binding of proteins on IEX membranes. It can also be crucial during the elution of proteins by the electrostatic interactions through increasing the ionic strength [40]. The maximum IgG binding capacity was reached at the lowest ionic strength (0 M KCl), and IgG binding capacity decreased when the ionic strength increased [71]. This is because the Debye length decreases as ionic strength increases, leading to a screening of the electrostatic forces. The Debye length is inversely proportional to the square root of the ionic strength of the solution, which is determined by the concentration and the valency of the ions present [72,73]. The ionic radius of different ions, such as LiCl, NaCl, KCl, and MgCl_2_, can affect the adsorption capacity of the membranes. With an increasing ionic radius, the adsorption capacity of lysozyme gradually decreased, possibly due to stronger shielding effects of ions with larger radii. A similar decrease in the adsorption capacity of the EVOH/BTCA nanofibrous IEX membrane was observed across the same ions [58]. The order of the adsorption capacities was LiCl > NaCl > KCl > MgCl_2_, which is consistent with the findings reported in reference [74] for the same protein, lysozyme. Similarly, ions with larger radii caused stronger shielding effects, hence reducing the binding capacity. Another study highlighted that as the ionic strengths of buffer solutions significantly influences the electrostatic interaction between nanofibre and biomolecules, as the Na_2_SO_4_ concentration increased from 0.1 to 0.4 M, the adsorption capacity sharply decreased [31]. This resulted from the gradually enhanced shielding effect on the electrostatic interaction between the IEX nanofibre membrane and lysozyme molecules. Meanwhile, adsorption performance can be further tailored by adjusting buffer properties and protein concentrations. This emphasises the importance of identifying the optimal use for practical application.

## 3. Modelling Approaches for Protein Binding

**Figure 1:**
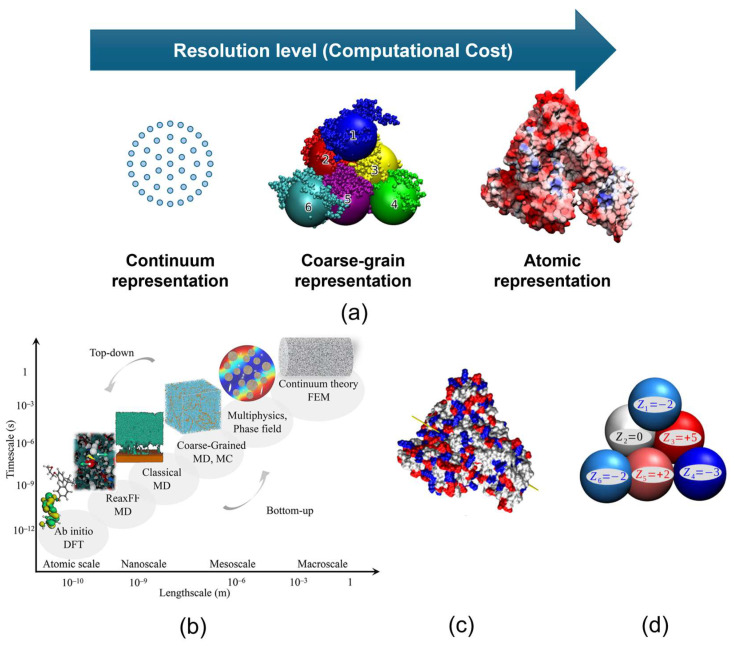
(**a**) Hierarchical classification of computational protein models by resolution level with BSA molecule; (**b**) a schematic representation of temporal and spatial scales; (**c**) distribution of charge on the BSA molecule surface coloured by residues’ total charge: positive—blue, negative—red, neutral—white; (**d**) coarse-grained charge distribution of BSA at its isoelectric point, expressed in units of the elementary charge; *e* (redrawn from [76,77,78,79]) reproduced from Ref. [76] with permission from the Royal Society of Chemistry.

Protein-binding models and computational tools vary substantially in their level of protein representation and in the transport phenomena they are designed to investigate. Due to the multi-scale nature of protein stability, it is computationally infeasible to employ a single model to address both nanometre-scale phenomena (e.g., conformational changes, protein–protein interactions) and macroscopic processes (e.g., aggregation, phase separation). Approaches to studying protein interactions can be classified as atomistic models, coarse-grain models, and continuum models [75]. Figure 1 illustrates these models and their time and length scales.

**Figure 2:**
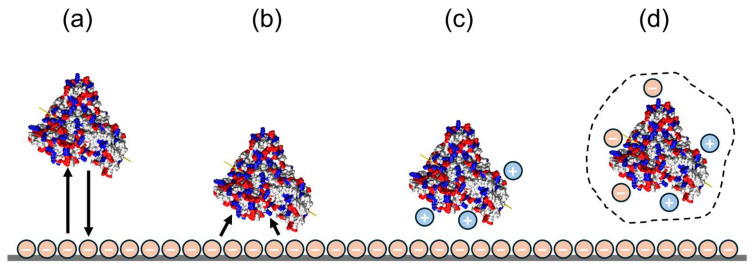
Commonly used binding models in ion-exchange: (**a**) the Langmuir model; (**b**) the stoichiometric model; (**c**) the Steric Mass Action (SMA) model; (**d**) the colloidal particle adsorption model. The BSA molecule is shown with its surface charge distribution coloured by the total charge of individual residues (blue: positive, red: negative, white: neutral). The “+” and “−” symbols represent mobile ions in the electrolyte surrounding the protein; the arrows illustrate electrostatic interactions with the surface under each modelling framework (redrawn from Ref. [92]).

In atomistic models, each atom is represented as a single bead and an individual interaction site (Figure 2a). The physical properties (mass, charge, etc.) of atoms and the behaviour of the protein solution are described through the potential energy function [76]. This function incorporates bonded interactions such as bond stretching, angle bending, and intra- and intermolecular non-bonded interactions such as electrostatic, van der Waals forces, and hydrogen bonding across all molecular species. Molecular dynamics (MD) and Monte Carlo simulations can be used to give molecular insights into protein adsorption to understand the mechanisms and driving forces behind surface interactions, conformational changes, and adsorption kinetics at the atomic level [77]. Monte Carlo (MC) simulation employs repeated random sampling to produce numerical results and is particularly useful for systems with high degrees of freedom that are challenging to model using other methods. While Density Functional Theory (DFT) and molecular dynamics (MD) are widely applied, linking atomic-scale data to experimentally observable macroscopic properties remains a significant challenge. In such cases, coarse-grained (CG) and dissipative particle dynamics (DPD) methods are employed to investigate interactions at the mesoscale [48].

Coarse-grained models are used when the degrees of freedom of a molecule are reduced by grouping atoms into lumped/coarse-grained beads [78]. In addition to these models, hybrid approaches can be employed to integrate the strengths of atomistic and coarse-grained models while eliminating their limitations [78]. In CG models, larger time and length scales can be simulated by the incorporation of fewer interaction sites [79]. In DPD simulations, particles interact via a soft repulsive potential that permits overlap between non-bonded particles. Consequently, oppositely charged point species may readily form artificial ion pairs. To avoid this, a common approach is to smear the charges of DPD particles using spatial distributions [80]. Coarse-grained molecular models can also be defined on a lattice [81]. The Lattice Boltzmann Method (LBM) models fluid flow by tracking the evolution of particle distribution functions over a fixed spatial lattice, as governed by the discrete Boltzmann equations [82]. This method is a mesoscopic method that bridges the gap between continuum-based methods and molecular dynamics by modelling fluid behaviour at an intermediate scale [83]. Computational modelling becomes crucial as it can provide thorough molecular-level information at a scale that can be difficult to observe experimentally [84].

Continuum models offer a robust alternative for simulating protein adsorption that occurs on time and length scales beyond seconds and micrometres, which are inaccessible to atomistic and coarse-grained approaches [75]. The adsorption equilibrium of macromolecules on a porous material is generally represented using empirical or semi-empirical models, such as the Langmuir isotherm, the distributed pore model, or the Steric Mass Action (SMA) law, each offering varying levels of efficiency [85]. While these models can predict the salt effect, they can lead to unreliable results at high protein concentrations. Compared to other adsorption isotherms, the SMA model offers the advantage of explicitly incorporating counterion concentration while capturing both linear and nonlinear adsorption behaviours [86]. The SMA model employs stoichiometric binding theory by coupling the system’s electrostatic and equilibrium characteristics within a set of correlation parameters [87]. Nonlinear adsorption behaviour up to adsorber saturation is explained by a reduction in available counter-ions on the adsorber surface. This occurs as adsorbed proteins either displace counter-ions or sterically shield them because of their size. Therefore, the model attributes adsorption limits not to the physical surface area of the adsorber but to its ionic capacity.

### 3.1. Modelling Frameworks for Ion-Exchange Protein Binding

Building on the existing protein-binding models, ion-exchange systems can also be analysed across different modelling scales, from molecular-level descriptions of electrostatic interactions to the macroscopic level used for parameter estimation and process design. Figure 2 shows the common adsorption models of protein adsorption on the IEX adsorber [88]. While stoichiometric adsorption models, such as the SMA model, offer a straightforward framework for describing protein adsorption, their capacity to accurately capture the electrostatic interactions between proteins and adsorbers is often debated [89]. Additionally, the SMA model requires prior information on three parameters for each macromolecule in solution: the characteristic charge, the equilibrium constant, and the steric factor. These parameters can be obtained through time- and resource-consuming chromatographic experiments involving gradient elution and breakthrough columns. As one of the challenges of these models, protein–protein interactions cannot be fully understood [90]. The theoretical limitations of stoichiometric models in describing electrostatic interactions between charged proteins and the charged adsorber surface, particularly concerning ionic strength and pH, have led to the creation of non-stoichiometric adsorption models [75].

CFD has been used to examine how membrane structural features, such as channel tortuosity, pore size, and connectivity and device design, have a significant influence on fluid flow distribution, as poorly engineered configurations can lead to substantial flow maldistribution that diminishes binding performance [55,91]. CFD tools enable the optimisation of membrane structures by modelling fluid dynamics, thereby informing the design of architectures that promote improved flow uniformity and performance. CFD simulations offer high-resolution flow fields around fibres and can be integrated with particle dynamics models, such as the discrete phase model (DPM) and discrete element model (DEM), to enable detailed analysis of particle permeation and behaviour near the fibres [92]. With CFD–DPM (discrete phase model) simulations, it is possible to track particles within the fluid medium and to study deposition behaviour of these sub-micron particles [93]. The CFD–DEM simulations predict colloid trajectories by computing the net force on each particle, incorporating hydraulic forces from the surrounding fluid flow, which can be gained through coupling with CFD [94]. Thus, using CFD coupled with DEM (discrete element method) would enable us to study each particle interaction with the surface as well as with the other particles and the surrounding medium [95]. Also, DEM simulations can simulate the particle interaction in the range of 10^6^–10^8^ particles [96]. The population balance model (PBM) simulations can predict the particle size changes in bulk-level growth to simulate particle aggregation during binding [97]. CFD–PBM (population balance model) simulations enable the evaluation of population dynamics governed by nucleation, growth, dispersion, dissolution, aggregation, and breakage by studying particle size distribution (PSD) on surfaces [98]. PBM and DEM are capable of capturing aggregation dynamics of particles, with PBM focusing on population-level characteristics through statistical approaches and DEM resolving particle-level interaction in detail [99]. In contrast, DPM cannot provide key information such as collision frequency and aggregation effects, limiting its suitability for studying particle aggregation [100]. Additionally, in DPM, the particles are treated as a dimensionless point mass; therefore, monitoring the distance between the surfaces of the particle and fibre is necessary during the trajectories [101]. While particle–particle (protein–protein) interactions during binding can be studied with DEM, DPM, and PBM simulations, it is not possible to study these interactions, which can limit their suitability for IEX binding prediction fully.

To improve the IEX binding and to design new high-throughput adsorptive membranes, the adsorption process needs to be understood. However, since the process occurs on a molecular level, it is not possible to observe the process through experimental techniques. These alternative models offer a more fundamental understanding of electrostatic interactions in ion-exchange (IEX) chromatography by using an idealised colloidal representation of the protein and analytical solutions to the linear Poisson–Boltzmann equation [102]. Theoretical modelling is required to obtain precise analytical expressions for the interaction potential essential for deducing nanoparticle properties such as size or charge and for computing phase behaviour or transport coefficients [48].

### 3.2. Application of DLVO Theory to Protein Binding on IEX Nanofibres

**Figure 3:**
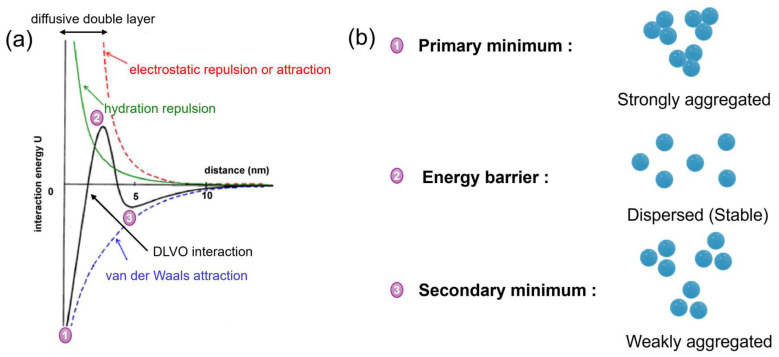
(**a**) The occurrence of three interaction forces: when a protein molecule approaches a surface, electrostatic attraction or repulsion, van der Waals attraction, and hydration repulsion; (**b**) description of the stability of colloidal particles (redrawn from Ref. [109]).

Colloidal interactions are often described by the DLVO theory, which is named after Derjaguin and Landau and Verwey and Overbeek [103]. DLVO theory offers a conceptually straightforward and accurate framework for describing interactions between surfaces separated by a liquid, playing a crucial role in understanding colloidal system behaviour [104]. DLVO theory attributes surface interactions to the combined effects of van der Waals forces and electrostatic double-layer interactions (Figure 3a).

At the primary minimum, interparticle interaction is strongest, leading to an unstable suspension characterised by strong aggregation or irreversible aggregation. At the energy barrier, repulsive forces dominate, resulting in a stable dispersion. The secondary minimum reflects weaker attraction, allowing for reversible or weak aggregation (Figure 3b) [105]. The energy barrier height increases with decreasing ionic strength, increasing zeta potential, and larger particle size. Conversely, the secondary minimum increases with higher ionic strength and particle size but lower zeta potential [60]. The DLVO model was used to predict the structural behaviour of lysozyme solutions under various conditions of pH and ionic strength [106]. It was found that DLVO theory generally captures the trend of protein interactions becoming increasingly short-range and attractive with rising pH and ionic strength. The DLVO theory also used another study to describe the interaction between BSA and PES using zeta potential, surface charge density, and interaction energy [107]. In the study, a secondary minimum of the interaction energy governed the binding of BSA on PES. Additionally, the interaction energy decreased with increasing ionic strength, while adsorption increased as the system pH decreased.

Besides DLVO theory, extended DLVO (xDLVO) theory was also used through the surface energetics approach to explain the interaction behaviour of proteins with IEX chromatographic supports [59]. According to xDLVO theory, protein interactions are governed by the combined effects of Lewis acid-base, van der Waals, and coulombic type forces [108]. xDLVO theory was used to explore the parameters used for the surface energetics approach to calculate the interaction energy minimum of different proteins binding onto three different supports, e.g., glass, plastic, and membrane [108]. The interaction behaviour is significantly influenced by ligand type (phenyl vs. butyl), ligand density (25–75%), and lambda conditions (0.2–1.0 nm). The surface energy components can be calculated from two experimental measurements: the contact angle, which helps to determine the Lifshitz van der Waals and acid-base components, and the zeta potential, which is used to determine the electrostatic component of surface energy [59].

### 3.3. Dynamics of Fibre–Protein Interactions

Understanding the interactions of proteins with the surfaces is based on the interaction energies between the proteins and the fibre surface. Interaction energy calculations can be commonly used to predict situations as either favourable or unfavourable for colloid aggregation. Protein binding on a surface is a complex process influenced by (1) protein properties, e.g., conformation, charge distribution, the strength of intramolecular bonds; (2) surface properties, e.g., chemistry, charge, roughness, surface energy; and (3) solution conditions, e.g., pH, salt concentration [109,110]. Because of these factors, the interactions are considered dynamic processes that theoretically involve several potentially interchangeable steps [111].

Surface morphology, besides the electrostatic interactions, can alter protein binding on surfaces [112]. The interaction between the colloid and surface is highly dependent on the nanoscale surface roughness properties of both surfaces. The influence of nanoscale roughness is likely to change with the solution chemistry, the colloid size, the surface charges, and the water velocity [111]. Roughness is a term used to characterise surface irregularities. The artificial modification of surface roughness was achieved by increasing the friction coefficient [113]. The interactions between particles and fibres were described in terms of friction and restitution coefficients. Friction between particle and fibre was found to influence particle behaviour during impact and adhesion [114]. The increase in the particle–wall friction coefficient led to an increase in the charge magnitude of the surface, as this allows a longer contact duration with the surface, causing more opportunities for charge transfer [115]. Most colloidal particles in suspension (0.001–10 μm) tend to remain suspended and settle very slowly in solution to maintain electrical neutrality due to the presence of surface charge arising from isomorphic substitution, chemical reaction at the interface, or preferential adsorption of ions on the particle surface from the surroundings by excess of oppositely charged ions (counterions). The combined interactions of this system of oppositely charged clouds are known as the electric double layer. The developed electrostatic repulsive forces prevent particle aggregation in the colloidal system and, hence, contribute to their stability in the dispersed system [116].

Surface energy is another important property that influences the interaction of a protein with the surface. Since proteins are charged molecules, a charged surface will impact their interactions [117]. At uncharged fluid interfaces, hydrophobic interactions primarily govern the binding behaviour, whereas at charged surfaces, a combination of hydrophobic and electrostatic interactions becomes more significant [84]. Hydrophobic surfaces have the advantage of interacting with hydrophobic residues of proteins, and water displacement from the surface is more favourable than on hydrophilic surfaces. On the other hand, the hydrophilic membranes were generally found to be more advantageous due to their possibility of reducing non-specific binding [1]. Wettability of surfaces not only influences the total amount of proteins but also their spatial conformation. For example, it was shown that the impact of surface wettability on protein confirmation during adsorption for bovine serum albumin (BSA) demonstrates that proteins adsorbed on more hydrophobic materials present a higher degree of denaturation. Usually, hydrophobic materials are non-polar, while hydrophilic substrates present a distribution of charges on their surfaces [118].

### 3.4. Influence of Protein–Protein Interactions During Binding

Protein–protein interactions can lead to challenges, such as aggregation, liquid–liquid phase separation, and increased solution viscosity, particularly at high protein concentrations [119]. The aggregation tendency of proteins can be minimised by controlling several factors such as temperature, pH, ionic strength, and protein concentration. When proteins are more likely to form partially folded states that lead to aggregation, a high net charge reduces their tendency to aggregate. This can cause repulsive double-layer forces to increase the colloidal stability of the protein. In solutions close to the protein’s isoelectric pH (pI), strong and attractive electrostatic interactions between proteins have been associated with enhanced aggregation tendencies [120]. Proteins at their pI lack net charge, weakening electrostatic repulsion [119]. The reduction in the net energy barrier for protein–protein interaction led to a loss of stability, thereby enhancing aggregation [121]. The aggregate binding mechanism is predominantly driven by electrostatic interactions [122].

Colloidal protein–protein interactions, including attractive and repulsive nature, can be decisive in protein’s solubility, aggregation, precipitation, and crystallisation. These interactions alone were not possible control or interfere with through observations or examinations during the experiments. Hence, protein–protein interactions are commonly quantified using the osmotic second virial coefficient (B_22_) or the diffusion interaction parameter (k_D_) [120]. There are a few models, such as Monte Carlo and molecular dynamics, that can be used to estimate B_22_ [123]. Among these models, DLVO theory can also be used to calculate B_22_ coefficients to better understand protein–protein interactions to optimise process conditions towards the stability of proteins [124]. Normally, the extended DLVO (xDLVO) model is used to describe B_22_ in aqueous solution as a function of pH, salt type and concentration, and temperature by fitting to experimental B_22_ data [124]. There are available coarse-grained simulations to quantify the binding of proteins on the surfaces. Since the hardship of quantification of such interactions, extended DLVO theory (xDLVO) can be used with a coarse-grained model (xDLVO-CG) to extend the existing models by a coarse-grained representation of protein and the inclusive additional ion–protein dispersion interaction term [124].

Generally, the adsorption of protein increases with the surface charge, but if the surface charge is too high, protein adsorption decreases due to the flat orientation of the protein. When increasing the charge of the surface, the adsorbed mass of a given charged protein increases. However, the adsorption of protein is very low if the charge on the surface is very high, probably due to the flat orientation of the protein [125]. A simplified protein–protein interaction model was based on a Baxter adhesive potential and an electric double-layer force to distinguish the contributions of longer-ranged electrostatic interactions from short-ranged attractive forces. The DLVO theory was successfully adopted to accurately capture the dependence of the ionic strength for solutions at pH 6.5 and below [119].

**Figure 4:**
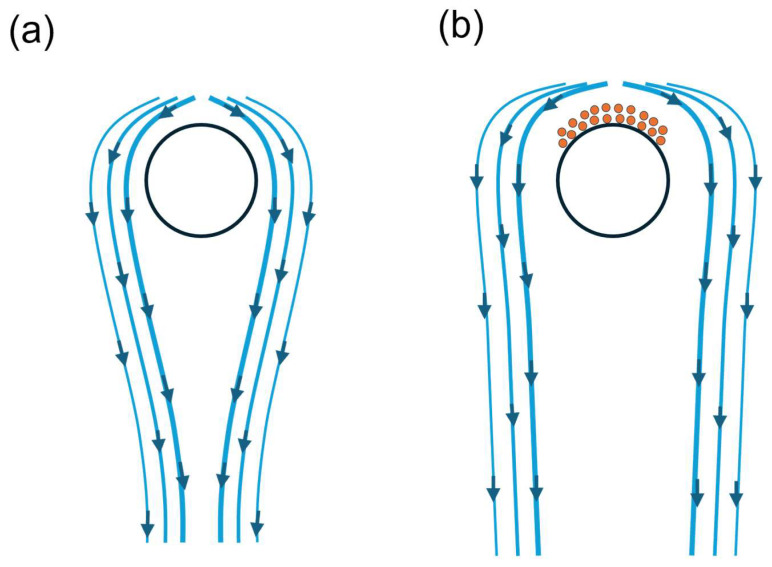
(**a**) Schematic representation of flow field around the fibre without discrete particles; (**b**) changed flow field due to deposited/bound particles on the fibre. The arrows represent the flow direction around the fibre. (redrawn from Ref. [121]).

Multilayer adsorption is dependent on both the protein type and concentration. For example, lysozyme binds to the surface due to its strong positive surface charge, showing higher electrostatic interaction than ɑ-chymotrpsin. In contrast, ɑ-chymotrpsin can form multilayer binding in both low and high concentrations, facilitated by its heterogeneous charge distribution and limited surface area of adsorptive surface availability [85]. Protein deposition on the surface of the fibre could change the dynamics of the adsorption and corresponding governing equations, as shown in Figure 4.

Therefore, it is crucial to simulate the protein–protein interactions along with the protein–surface interaction for studying the adsorption of proteins on electrospun nanofibres. Binding energies can be studied in detail while accounting for van der Waals and electrostatic repulsion (DLVO) forces between the proteins and the fibre surface. Additionally, the relationship between binding and membrane properties can also be studied in detail.

## 4. Conclusions and Future Perspectives

Colloidal and meso-scale modelling approaches provide a powerful tool “sweet spot” for analysing protein binding mechanisms, offering sufficient resolution to capture key interaction dynamics with no need for the computational cost of fully atomistic simulations. These methods bridge molecular interactions with macroscopic binding properties enabling detailed visualisation and quantification of how pore geometry, surface charge distribution, and local hydrodynamics influence protein transport and binding within nanofibrous or porous membranes.

Protein binding is influenced by numerous factors, including adsorbent properties, protein characteristics, and solution conditions. Experimentally evaluating each of these individually can be time-consuming and resource-intensive. Therefore, computational approaches allow systematic exploration of these parameters, providing predictive insights into their effects on binding behaviour. When combined with theoretical frameworks such as DLVO-based interaction energy calculations, they offer predictive insight into protein–fibre interactions, binding propensity, and competitive adsorption effects under different solution conditions. By capturing interplay between electrostatics, steric constraints, and convective transport, such models directly support rational membrane design by identifying optimal surface chemistries, pore architectures, and operating conditions required to maximise binding efficiency, selectivity, and overall chromatographic performance.

Future membrane fabrication for chromatographic applications should prioritise optimising the pore structures of nanofibrous membranes to enhance adsorption performance. Understanding pore architecture and surface modification effects on mass transport is essential for evaluating membrane binding performance. Overall, the concepts and modelling frameworks summarised in this work not only enhance our understanding of protein binding in membrane chromatography but also extend to broader applications in the design of protein purification and antifouling membranes, where understanding protein–surface interactions is crucial. This broader relevance highlights the wider impact of the modelling frameworks and their potential to support future advances across membrane-based separation technologies. Future research should extend these modelling frameworks to more accurately represent flexible nanofibre systems, where dynamic processes can be captured. Adopting colloidal-scale approximations of proteins offers a computationally efficient yet physically meaningful route to study binding dynamics on nanofibrous materials. By representing proteins as soft colloidal particles, such models can capture essential features of adsorption and transport without the prohibitive cost of fully atomistic simulations. This approach enables systematic exploration of critical process parameters such as surface charge density, pore connectivity, and flow conditions that govern binding performance. Consequently, colloidal-level modelling can accelerate and guide the design of high-throughput and cost-effective ion-exchange membranes by providing insights that bridge molecular detail with process-scale functionality.

The future of IEX-NFM design lies in the convergence of physics-based modelling and data-driven intelligence. Machine learning models trained on experimental and simulated datasets could rapidly predict protein-binding capacity, pressure drop, and mass transport behaviour from structural inputs such as fibre morphology and properties [126]. Coupled with material informatics approaches for feature selection and design, this will allow accelerated optimisation of membrane architectures. In the long run, integrating AI-guided design and high-throughput characterisation could establish continuous improvement of membrane materials [127].

## Figures and Tables

**Table 1:** Protein separation by various modes and their mechanisms.

Separation Type	Method
Ion exchange	Reversible adsorption of a charged protein to ion-exchange matrices containing oppositely charged covalently attached side groups [4]
Hydrophobic interaction	Adsorbents, which have covalently attached hydrophobic groups Hydrophilic region exposure is promoted by decreased ionic strength [12]
Affinity	Specific interaction between the immobilised ligand and the binding site on the target molecule [13] Selective and efficient target molecule capturing [14] Most selective chromatography types among others [12]
Mixed-mode	Selective protein adsorption by the combination of several separation methods and membrane adsorbents [8]

**Table 2:** Performance of nanofibrous membranes in MC for the purification of targeted proteins across various studies. “*” is for the unit of mg/mL. Unless it is marked “*”, it is mg/g.

Ref.	Nanofibrous Membrane	Fibre Diameter (d_f_) (nm)	Water Contact Angle (WCA) (^o^)	Mean Pore Size (µm)	Specific Surface Area (SSA) (m^2^/g)	Target Protein	pH	Initial Concentration (mg/mL)	Binding Capacity (mg/g or mg/mL *)
[35]	PAN-pAQ	353 ± 69–673 ± 36	115 ± 5–132 ± 0.6	0.9 ± 0.1–1.7 ± 0.2	4.4–9.3	BSA	7.5	-	98 ± 8–166 ± 2 (SBC)
[36]	PSf-GMA-DEA PAN-GMA-DEA	2500 ± 460 (PSf) 150–340 (PAN)	128 ± 4.8 (PSf) 73.1 ± 3.5 (PAN)	-	-	BSA	7.0	0.5–3	201.3 ± 4.8 * (PSf) 87.2 ± 2.6 * (PAN) (DBC) 260 * (PSf) 100 * (PAN) (SBC)
[16]	EVOH-CCA	562	120	-	2.52	Lysozyme	4–10	1	250 (DBC)
[37]	SA-PEO	150	-	0.3–0.6	13.56 (g/m^2^)	Lysozyme	3.18	0.4–2.0	805 (DBC) 1235 (SBC)
[38]	CA-DEAE and CA-COO	500	-	-	-	Lysozyme BSA	8.0/BSA5.5/Lysozyme	2.0	COO-27 * lysozyme (DBC) & DEAE-20 * BSA (DBC)
[39]	PVA-MAH	226-(30% PVA) 284-(5% PVA)	45	0.002–0.064	3.2–3.5 (g/m^2^)	Lysozyme	6.0	0.1–1.2	159 (DBC)
[40]	CMA	267	124	0.65	3.28 (g/m^2^)	Lysozyme	4–8	1	160 (SBC) 118 (DBC)

## Data Availability

No new data were created or analyzed in this study. Data sharing is not applicable to this article.
